# Pleomorphic adenoma and carcinoma ex‐pleomorphic adenoma tumorigenesis: A proteomic analysis

**DOI:** 10.1111/odi.15109

**Published:** 2024-08-18

**Authors:** Virgílio Gonzales Zanella, Sara Ferreira Dos Santos Costa, Lauren Frenzel Schuch, Emily Ferreira Salles Pilar, Adriana Franco Paes Leme, Jean Nunes dos Santos, Syed Ali Khurram, Fatima Elalawy, Lynne Bingle, Fabio Daumas Nunes, Felipe Paiva Fonseca, Pablo Agustin Vargas, Manoela Domingues Martins, Vivian Petersen Wagner

**Affiliations:** ^1^ Department of Pathology, School of Dentistry Federal University of Rio Grande do Sul Porto Alegre Brazil; ^2^ Head and Neck Surgery Department Santa Rita Hospital, Santa Casa de Misericórdia de Porto Alegre Porto Alegre Brazil; ^3^ Department of Oral Surgery and Pathology, School of Dentistry Federal University of Minas Gerais Belo Horizonte Brazil; ^4^ Department of Oral Diagnosis, Piracicaba Dental School University of Campinas Piracicaba Brazil; ^5^ Experimental Research Unit Hospital de Clínicas Porto Alegre Porto Alegre Brazil; ^6^ Postgraduation Program in Dentistry and Health Federal University of Bahia Salvador Brazil; ^7^ Academic Unit of Oral and Maxillofacial Medicine and Pathology, Department of Clinical Dentistry University of Sheffield Sheffield UK; ^8^ Department of Oral and Maxillofacial Pathology, Dental School University of São Paulo (USP) São Paulo Brazil

**Keywords:** carcinoma ex‐pleomorphic adenoma, glandular and epithelial, head and neck neoplasms, proteomic, rare diseases

## Abstract

**Objectives:**

To conduct a comprehensive proteomic analysis of normal salivary gland tissue, pleomorphic adenoma (PA), and carcinoma ex‐pleomorphic adenoma (CXPA), and validate the proteomic findings using immunohistochemistry.

**Methods:**

Six normal salivary gland tissues, seven PA and seven CXPA samples underwent laser microdissection followed by liquid chromatography coupled to mass spectrometry. Protein identification and quantification were performed using MaxQuant software. Statistical analysis and functional enrichment were conducted using the Perseus platform and STRING tool, respectively. Immunohistochemistry was used for validation.

**Results:**

Comparative proteomic analysis revealed 2680 proteins across the three tissue types, with 799 significantly altered between groups. Translocation protein SEC63 homolog, Annexin A6 and Biglycan were up‐regulated in CXPA compared to PA. Decorin was markedly up‐regulated in both PA and CXPA compared to normal salivary gland (log_2_ fold changes of 7.58 and 7.38, respectively). Validation confirmed elevated levels of Biglycan and Decorin in the extracellular matrix of CXPA compared to PA.

**Conclusions:**

Proteomic analysis identified differential protein expression patterns associated with malignant transformation of PA into CXPA. Findings indicate a crucial role for extracellular matrix proteins, specifically Biglycan and Decorin, in the tumorigenic progression of PA and CXPA.

## 1 INTRODUCTION

Pleomorphic adenoma (PA) is the most common salivary gland neoplasm (da Silva et al., 2018; Sentani et al., 2019; Vasconcelos et al., 2016) which in some cases may undergo malignant transformation to carcinoma ex‐pleomorphic adenoma (CXPA). The reported rate of malignant transformation of primary PA is 6.2% (ranging from 1.9% to 23.3%). The epithelial and/or myoepithelial component can transform into CXPA, which is an uncommon tumor making up just 3%–15% of cases of salivary gland malignancies (Altemani et al., 2005; Andreasen et al., 2016; Di Palma, 2013; Ferreira et al., 2014; Lüers et al., 2009; Skálová et al., 2022; Valstar et al., 2017; Zanella et al., 2021; Zbären et al., 2008). The biological mechanisms involved with PA development and malignant transformation are currently not well understood but recent studies have started to elucidate this process (de Lima‐Souza et al., 2023; Scarini et al., 2023; Valstar et al., 2020).

Proteomics consists of the large‐scale study of the proteins expressed in a sample at a given time, which allows the detection of differential protein expression profiles, protein modifications, and protein–protein interactions (Liang et al., 2009; Mann, 2006). This global protein profile analysis has been proven to be an important tool in identifying potential markers for diagnosis, prognosis, and new targets for treatment of many diseases. Mass spectrometry measures a large number of unknown proteins in a sample through the chemical and physical separation of ions and by determining their mass to charge ratio (*m/z*) (Sparkman, 2000). It has previously been applied to tissue samples, saliva, plasma, and cell lines in attempts to identify potential biomarkers of head and neck cancer (Gallo et al., 2016; Jarai et al., 2012; Ralhan et al., 2011). However, proteomic data on PA and particularly the transformation to CXPA are limited with only a few studies having utilized untargeted proteomic analysis. One such study focused solely on PA (Mutlu et al., 2017), while another did encompass both PA and CXPA (de Lima‐Souza et al., 2023) but did not include normal salivary gland (NSG) tissue. Moreover, validation through immunohistochemistry was not conducted in either study (de Lima‐Souza et al., 2023; Mutlu et al., 2017).

The aim of this study was to perform a comparative proteomic analysis of normal salivary gland tissue, PA and CXPA. The most down‐regulated and most up‐regulated proteins in neoplastic samples compared to normal tissue were selected for further validation using immunohistochemistry. Our results contribute to the understanding of the process of PA malignant transformation and suggest that extracellular matrix (ECM) components may play a key role.

## 2 MATERIALS AND METHODS

### 2.1 Study flowchart

Samples from a Brazilian cohort underwent laser microdissection followed by liquid chromatography coupled to mass spectrometry. Subsequently, immunohistochemistry was conducted to validate the findings using the same Brazilian cases alongside a further 20 cases from the United Kingdom.

### 2.2 Study design and patients

This study was approved by the Ethics Committee on Human Research (CAAE no. 74754317.5.0000.5335). Formalin‐fixed paraffin‐embedded samples of six NSG tissues, seven PA and seven CXPA were used when the diagnosis was reviewed and confirmed by two experienced pathologists according to the latest WHO criteria (Skálová et al., 2022). NSG tissue was obtained from inflammatory reactive lesions that were diagnosed in our pathology service and comprised normal as well as inflamed salivary gland tissue. The PA and CXPA were not paired samples and originated from different patients.

### 2.3 Patient data collection and tumor analysis

Data regarding age, sex, and primary location were retrieved from the medical records with follow‐up information being retrieved when available. PA cases were classified as myxoid, conventional or cellularized, and the malignant area of CXPA was classified according to the tumor subtype.

### 2.4 Laser microdissection (LMD)

Eight micrometer sections were prepared for LMD within PEN glass slides. Samples were then dewaxed and stained with Harris hematoxylin for 2 min. LMD was performed with a Zeiss PALM MicroBeam Laser Micro‐dissector with PALMRobo software version V4.6.Ink Samples were micro dissected with an average of 1,200,000 μm^2^ and only neoplastic tissue was selected for enrichment. Representative areas were selected, with particular attention taken to collect more hypercellular areas.

### 2.5 Protein extraction and digestion

All samples were treated with 8 M urea for denaturation, 5 mM dithiothreitol for disulfide bond reduction (DTT, 25 min, 56°C), 14 mM iodoacetamide for alkylation of cysteine residues (IAA, 30 min, room temperature in the dark) and digested with trypsin (overnight, Promega, 1:50 w/w, 16 h, at 37°C) (Villén & Gygi, 2008). The reactions were stopped with 0.4% formic acid. Peptides were desalted using C18 Stage Tips and reconstituted in 0.1% formic acid.

### 2.6 Liquid chromatography coupled to mass spectrometry

For detection, identification and relative quantification of the proteins, high‐performance liquid chromatography coupled to high‐resolution tandem mass spectrometry (LC–MS/MS) was used.

The resulting mixture of peptides was analyzed on a LTQ Orbitrap Velos mass spectrometer (Thermo Fisher Scientific, Waltham, MA, USA) coupled to the EASY‐nLC nanoflow liquid chromatography system (Proxeon Biosystem, West Palm Beach, FL, USA) via a Proxeon electrospray ionization source. Peptides were separated by a gradient of 2%–90% acetonitrile for mobile phase into 0.1% formic acid for stationary phase using a PicoFrit capillary chromatographic analytical column (20 cm × ID75 μM, 5 μM particle size; New Objective, Woburn, MA, USA) with a flow rate of 300 nL/min for 212 min. The nanoelectrospray voltage was 2.2 kV, and the source temperature was 275°C.

Data acquisition was performed by data‐dependent acquisition (DDA). The full scan of MS spectra (*m/z* 300–1600) was acquired on the Orbitrap analyzer after accumulation with a target value of 1 × 10^6^. The resolution on the Orbitrap was *r* = 60,000 and the 20 most intense peptide ions with ≥2 charge states were sequentially isolated to a value of 5000 and fragmented into linear ion through low collision energy by collision‐induced dissociation (CID) with a normalized collision energy of 35%.

### 2.7 Protein identification and quantitative analysis

The results in raw file format were processed using MaxQuant software version 1.3.0.3 (Cox & Mann, 2008) and the MS/MS spectra were submitted to the Andromeda integrated peptide search engine (Cox et al., 2011) against the international protein sequence and functional information database UniProt (http://www.uniprot.org).

The precursor mass tolerance was 10 ppm for first search and 6 ppm for database searches in Andromeda. The mass tolerance for fragment ions was 0.5 Da. Enzyme specificity was for trypsin with tolerance of peptides with up to two undigested cleavage sites. Methionine oxidation (15.994915 Da) and N‐terminal protein acetylation (42.010565 Da) were defined as variable modifications and cysteine carbamylation (57.021464 Da) as a fixed modification. The minimum peptide length was seven amino acids. The false discovery rate (FDRs) for peptides and proteins was 1%. The relative abundance value was obtained for all proteins identified by the label‐free quantification method based on the normalized intensity of the spectrum (LFQ intensity, label‐free quantification).

### 2.8 Data annotation and statistical analysis

Statistical analysis was performed using the Perseus bioinformatics platform, version 1.2.7.4 (Cox & Mann, 2008). Initially, the abundance matrix entries were identified only by modification site and those identified by reverse sequence were excluded from further analysis. The relative abundance of each protein was then converted to log_2_. The entries in the abundance matrix were filtered to contain only two valid values in at least one group for the subsequent analyses. As keratin proteins are considered contaminants by the platform, but are of interest in the study of epithelial tissues, true contaminants were manually removed (Carnielli et al., 2018). The ANOVA test was used to identify proteins with statistically significant differences in abundance between the groups (*p*‐value < 0.05) and a Venn diagram constructed to demonstrate shared and unique proteins (Heberle et al., 2015).

### 2.9 Protein–protein interaction and functional enrichment analyses

Protein–protein interactions (PPI) were assessed using the STRING tool (Search Tool for Retrieval of Interacting Genes), version 10.5 (Szklarczyk et al., 2017). The confidence score set for the interactions was a value ≥0.4.

STRING was also used to analyze gene ontology (GO) using three categories, biological process, cellular component and molecular function. A pathway enrichment analysis using the Kyoto Encyclopedia of Genes and Genomes (KEGG) was also performed.

### 2.10 Immunohistochemistry

Two proteins were selected for further validation by immunohistochemistry on a total of 34 cases, which included the original 14 cases used for the proteomics analysis (seven PA and seven CXPA) and an additional 20 cases obtained from the Academic Unit of Oral and Maxillofacial Pathology at the University of Sheffield (NREC Ref number: 05/Q2305/127), comprising nine cases of PA and 11 cases of CXPA. Data regarding age, sex, and primary location were retrieved from pathological reports.

Also, 3 μm sections on silanized slides were incubated with primary antibodies overnight (Biglycan monoclonal, 1:500, clone 4E1‐1G7; Decorin monoclonal, 1:250, clone 3H4‐1F4). The sections were then incubated with secondary antibody and diaminobenzidine (DAB) tetrahydrochloride (Vectastain Elite ABC) and counterstained with Mayer's hematoxylin. The slides were scanned using a digital slide scanner (Aperio CS2, Milton Keynes, UK) and whole slide images (WSI) of PA and CXPA assessed using QuPath software (Bankhead et al., 2017). The percentage of stained area was investigated by thresholder pixel analysis using the following parameters: (1) resolution: full; (2) channel: DAB; (3) threshold: 0.25; (4) region: any (5) annotation: ROI. Manual annotation was performed to exclude normal surrounding tissue (Figure S1) and results compared using the Mann–Whitney *U*‐test (significance < 0.05) in GraphPad software, version 10.0 (GraphPad Software, San Diego, CA, USA).

## 3 RESULTS

### 3.1 Clinical and histological aspects of the samples

Seven cases of PA and seven cases of CXPA were included for proteomic analysis with the average age at diagnosis for PA and CXPA being 45.7 and 63.8, respectively, and the male:female distribution for PA and CXPA being 3:4 and 4:3, respectively. All cases used for proteomics analysis were primary parotid tumors.

The proteomic results, presented below, were validated using in total 16 cases of PA and 18 cases of CXPA (Table S1). The average age at diagnosis for the total sample of PA and CXPA were 47.2 and 62.8, respectively. All PA samples originated in the parotid gland while the CXPA samples comprised 13 cases (72.2%) from the parotid gland and 5 cases (27.7%) from other primary sites, including upper lip (*n* = 2), submandibular gland (*n* = 2), and parapharyngeal space (*n* = 1). PA were mostly classified as cellular (*n* = 6) and CXPA as adenocarcinoma not otherwise specified (*n* = 8) (Figure S2 and Table S1).

### 3.2 Comparative proteomic profile

**FIGURE 1:**
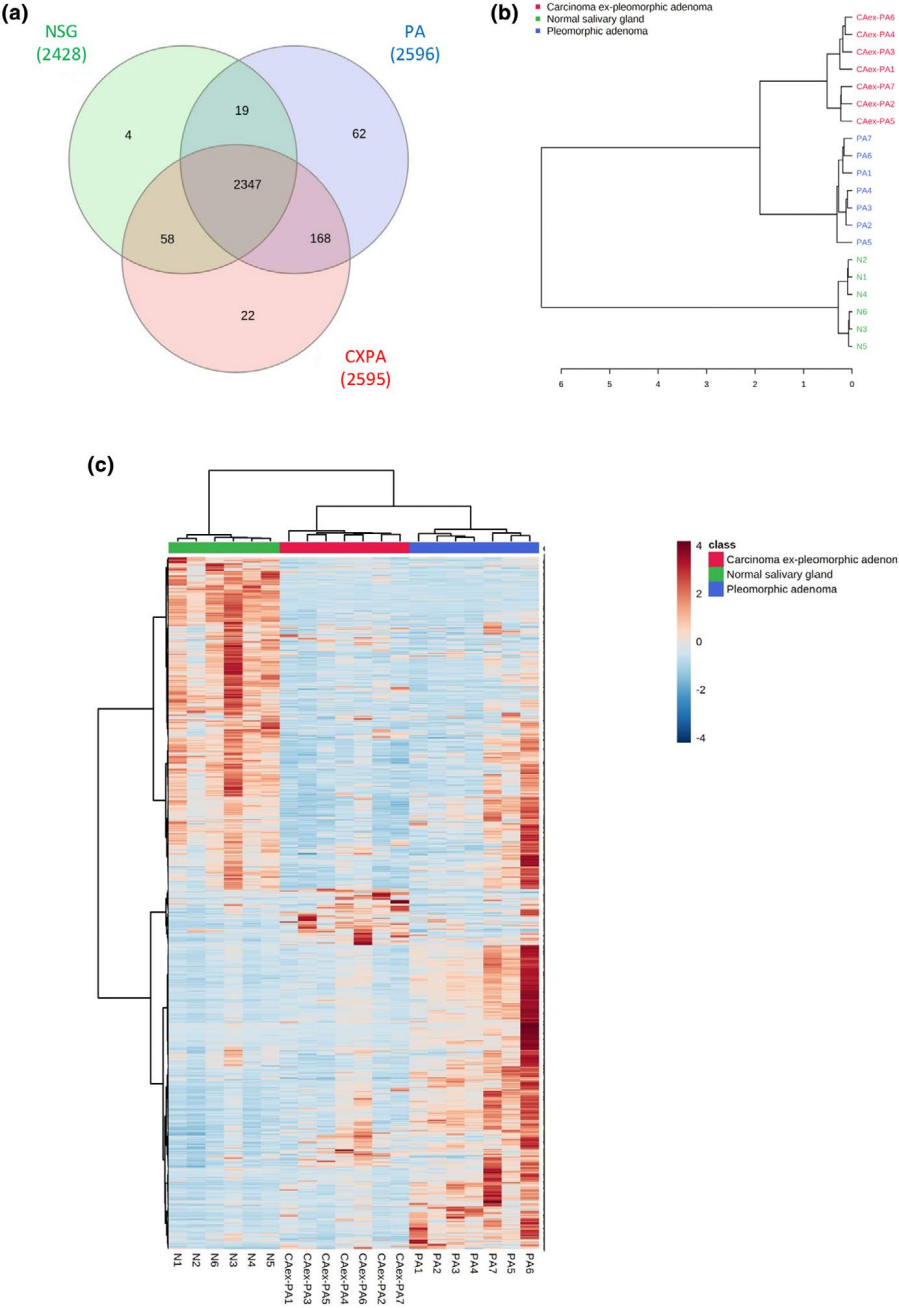
Proteins identified and quantified in the three groups. (a) Venn diagram of common and unique proteins in the three groups showed 2347 proteins identified in common in the three groups analyzed, 168 proteins shared between neoplasm groups, 58 proteins common between CA ex‐PA and NSG, and 19 proteins shared between PA and NGS. Of the total number of proteins identified, only 62, 22, and 4 were exclusively identified in PA, CA ex‐PA, and NSG, respectively. (b) In an exploratory analysis using a hierarchical method, the dendrogram of the proteins identified showed the hierarchy and clusters between the samples in each group. The cases analyzed are listed on the right vertical axis, whereas the horizontal axis shows the distance between the clusters when they are joined. The result indicated the initial formation of two clusters: The neoplasm cluster and the NSG cluster, with the CA ex‐PA and PA clusters being more closely correlated with each other and different from the NGS cluster. In addition, there was greater similarity between the samples in each group, demonstrated by the high correlation between cases in the same group. (c) The heat map was used to compare the abundance of proteins between the samples analyzed in the three groups (*Z*‐score log_2_ intensity values). Each row represents an identified protein and each column is a single sample analyzed. The intensity of the color at each intersection point indicates the level of abundance of each protein per sample, with high abundance shown in red and low abundance in blue. The cluster analysis clearly identified the main groupings as shown in the color bar at the top in green, red, and blue representing the NSG, PA, and CXPA groups, respectively.

Quantitative proteomic analysis resulted in the identification of 2680 proteins from the three tissue types investigated (Table S2). The distribution between the groups (shared and unique proteins) is shown in the Venn diagram (Figure 1a) with reproducibility analysis being assessed by correlation coefficient (Pearson *r*) among the log_2_ LFQ intensities (Table S3).

**FIGURE 2:**
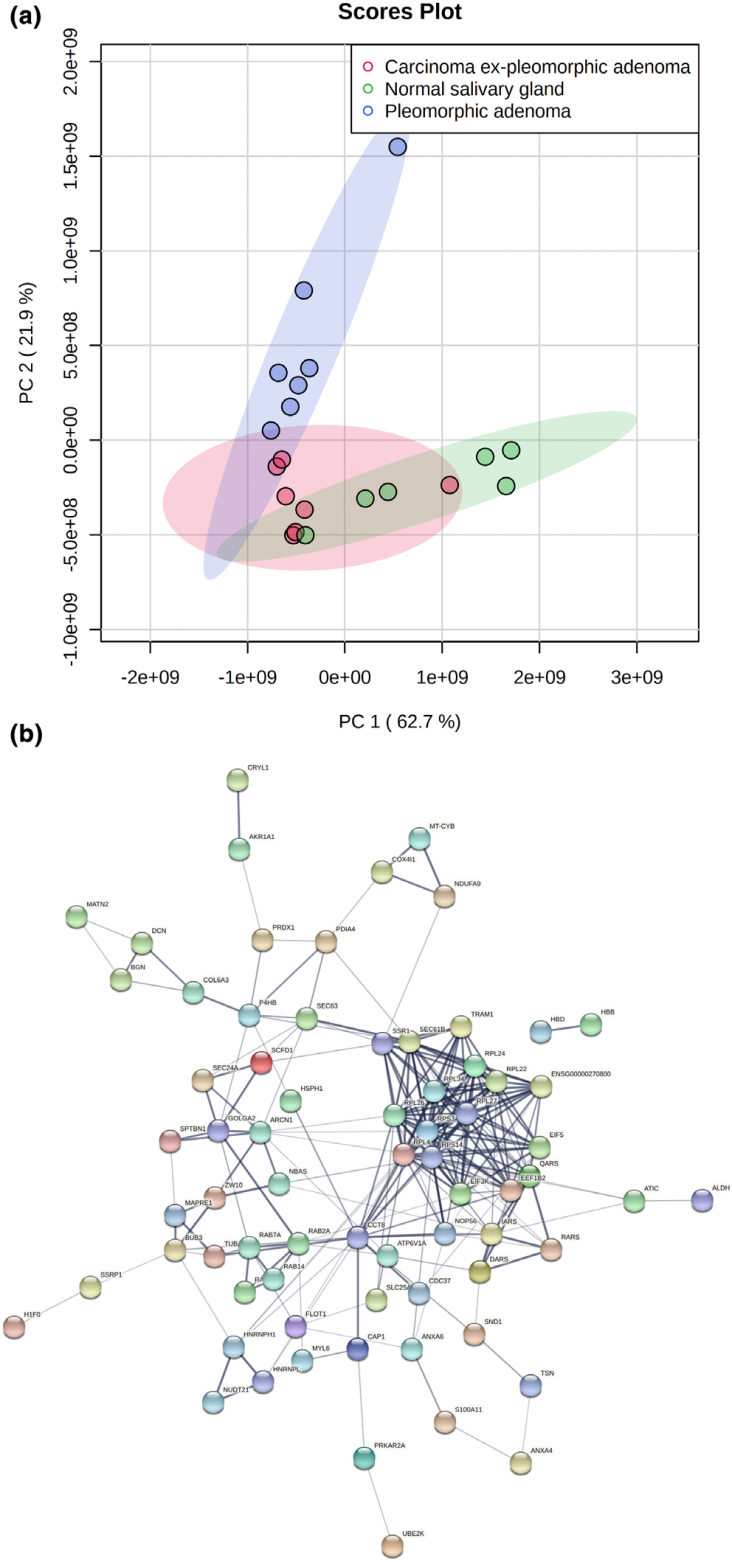
(a) Principal component analysis (PCA) presented as a score‐plot 2D of the main components observed among the three groups demonstrated the separation between samples according to abundance variation. The colored dots represent a single sample in each group. The samples in each group are close to each other forming three distinct groups. (b) Protein–protein interactions (PPI) networking showing groups of highly correlated proteins as indicated by the black traces (the thicker the trace, the stronger the correlation between the proteins).

The unsupervised hierarchical clustering analysis performed with the LFQ intensity values of the identified proteins demonstrated different proteomic profiles between the three groups analyzed, as represented in the dendrogram and heat‐map (Figure 1b,c). To evaluate the differences and similarities between the proteome of PA, CXPA and NSG, comparisons were made using a Principal Component Analysis (PCA) constructed with the total proteins identified. Separation among the groups was observed (Figure 2a) in that PA (blue area) and NSG (green area) clustered; the separation between these groups was more pronounced; however, there was some overlap between CXPA (red area) and the other groups.

A difference in the levels of 799 proteins was observed between groups (ANOVA test, Benjamini–Hochberg correction, adjusted *p* < 0.05; Table S4). Comparing PA to CXPA, NSG to PA, and NSG to CXPA, we found significantly different expressions of 39 (Table 1), 63 (Table 2), and 48 (Table 3) proteins, respectively. Decorin, a protein associated with the ECM, was the most up‐regulated protein of both benign and malignant tumors compared to NSG (log_2_ fold change of 7.58 and 7.38, respectively) while Biglycan, another ECM component, was the most down‐regulated protein in neoplastic samples compared to NSG (PA and CXPA log_2_ fold change of −9.96 and −7.29, respectively). Biglycan was also found to be significantly up‐regulated in CXPA compared to PA (log_2_ fold change −2.66) as were Translocation protein SEC63 homolog (4.40) and Annexin A6 (4.07).

**TABLE 1:** Up‐regulated proteins (log FC ≥ 2; *p* values adjusted <0.05) and down‐regulated proteins (log FC ≥ −2; *p* values adjusted <0.05) in carcinoma ex‐pleomorphic adenoma compared to pleomorphic adenoma.

	Protein name	Gene symbol	*p* Value	log_2_FC
Up‐regulated proteins	Translocation protein SEC63 homolog	*SEC63*	<0.001	4.40
	Annexin A6	*ANXA6*	0.002	4.07
	Hemoglobin subunit beta	*HBB*	0.003	3.70
	Calmodulin‐like protein 5	*CALML5*	0.009	3.23
	Aspartate–tRNA ligase, cytoplasmic	*DARS*	<0.001	3.21
	Coatomer subunit delta	*ARCN1*	<0.001	3.19
	Lambda‐crystallin homolog	*CRYL1*	0.002	2.77
	Mitotic checkpoint protein BUB3	*BUB3*	<0.001	2.69
	Biglycan	*BGN*	<0.001	2.66
	Alcohol dehydrogenase [NADP(+)]	*AKR1A1*	<0.001	2.64
	Ras‐related protein Rab‐2A	*RAB2A*	<0.001	2.42
	60S ribosomal protein L27	*RPL27*	<0.001	2.32
	UPF0762 protein C6orf58	*C6orf58*	<0.001	2.19
	60S ribosomal protein L10	*RPL10*	<0.001	2.15
	Golgin subfamily A member 2	*GOLGA2*	0.001	2.09
	cAMP‐dependent protein kinase type II‐alpha regulatory subunit	*PRKAR2A*	<0.001	2.00
	V‐type proton ATPase catalytic subunit A	*ATP6V1A*	0.003	2.00
	Flotillin‐1	*FLOT1*	0.01	2.00
Down‐regulated proteins	Protein transport protein Sec24A	*SEC24A*	<0.001	−4.12
	Protein NipSnap homolog 1	*NIPSNAP1*	<0.001	−3.72
	Mast cell carboxypeptidase A	*CPA3*	0.002	−2.81
	Hemoglobin subunit delta	*HBD*	0.002	−2.77
	Interferon‐induced guanylate‐binding protein 1	*GBP1*; *GBP2*	0.007	−2.77
	60S ribosomal protein L4	*RPL4*	<0.001	−2.75
	Elongation factor 1‐beta	*EEF1B2*	<0.001	−2.55
	Fermitin family homolog 2	*FERMT2*	0.009	−2.48
	Glutamine–tRNA ligase	*QARS*	<0.001	−2.44
	Aspartyl/asparaginyl beta‐hydroxylase	*ASPH*	<0.001	−2.40
	Dynamin‐1‐like protein	*DNM1L*	0.002	−2.36
	Microtubule‐associated protein RP/EB family member 1	*MAPRE1*	<0.001	−2.28
	Thymosin beta‐4; Hematopoietic system regulatory peptide	*TMSB4X*	<0.001	−2.25
	Dystrophin	*DMD*	0.001	−2.21
	Tax1‐binding protein 3	*TAX1BP3*	<0.001	−2.19
	Cleavage and polyadenylation specificity factor subunit 5	*NUDT21*	0.004	−2.16
	Ubiquitin‐conjugating enzyme E2 K	*UBE2K*	0.008	−2.13
	Arylsulfatase A; Arylsulfatase A component B	*ARSA*	<0.001	−2.11
	Arginine–tRNA ligase, cytoplasmic	*RARS*	<0.001	−2.08
	Protein transport protein Sec61 subunit beta	*SEC61B*	<0.001	−2.03
	Peroxiredoxin‐1	*PRDX1*	0.001	−2.01

**TABLE 2:** Up‐regulated proteins (log FC ≥ 2) and down‐regulated proteins (log FC ≥ −2) in pleomorphic adenoma compared to normal salivary gland tissue.

	Protein name	Gene symbol	*p* Value	log_2_FC
Up‐regulated proteins	Decorin	*DCN*	<0.001	7.58
	Dystrophin	*DMD*	0.001	6.88
	Protein transport protein Sec24A	*SEC24A*	0.0002	6.19
	Interferon regulatory factor 2‐binding protein‐like	*IRF2BPL*	<0.001	5.70
	Dynamin‐1‐like protein	*DNM1L*	0.002	4.81
	Ras‐related protein Rab‐14	*RAB14*	0.003	4.61
	Hsp90 co‐chaperone Cdc37	*CDC37*	0.003	4.05
	Tropomyosin alpha‐1 chain	*TPM1*	0.001	3.77
	Glutamine–tRNA ligase	*QARS*	<0.001	3.51
	Myosin light polypeptide 6	*MYL6*	0.001	3.46
	Peroxiredoxin‐1	*PRDX1*	0.001	3.38
	Tax1‐binding protein 3	*TAX1BP3*	<0.001	3.19
	Translocon‐associated protein subunit alpha	*SSR1*	<0.001	3.12
	Protein transport protein Sec61 subunit beta	*SEC61B*	<0.001	3.00
	Heterogeneous nuclear ribonucleoprotein L	*HNRNPL*	0.002	2.98
	Fermitin family homolog 2	*FERMT2*	0.009	2.94
	Bifunctional purine biosynthesis protein PURH	*ATIC*	<0.001	2.89
	Programmed cell death protein 4	*PDCD4*	0.003	2.70
	Squamous cell carcinoma antigen recognized by T‐cells 3	*SART3*	0.005	2.58
	60S ribosomal protein L22	*RPL22*	<0.001	2.518
	Cytochrome c oxidase subunit 4 isoform 1, mitochondrial	*COX4I1*	0.005	2.49
	Annexin A4	*ANXA4*	<0.001	2.48
	Mast cell carboxypeptidase A	*CPA3*	0.002	2.43
	Protein NipSnap homolog 1	*NIPSNAP1*	<0.001	2.40
	Long‐chain fatty acid transport protein 1	*SLC27A1*	0.001	2.35
	Tropomyosin beta chain	*TPM2*	0.009	2.35
	T‐complex protein 1 subunit theta	*CCT8*	0.002	2.21
	Ig mu chain C region	*IGHM*	0.014	2.21
	RPS10‐NUDT3	*RPS10‐NUDT3*	<0.001	2.19
	Collagen alpha‐3 (VI) chain	*COL6A3*	<0.001	2.17
	Aspartate aminotransferase, cytoplasmic	*GOT1*	<0.001	2.14
	Matrilin‐2	*MATN2*	<0.001	2.13
	Ras‐related protein Rap‐1b	*RAP1B*	<0.001	2.13
	Protein disulfide‐isomerase A4	*PDIA4*	<0.001	2.11
	Aldehyde dehydrogenase family 16 member A1	*ALDH16A1*	0.009	2.09
Down‐regulated proteins	Biglycan	*BGN*	<0.001	−9.96
	Adenylyl cyclase‐associated protein 1	*CAP1*	<0.001	−5.53
	Staphylococcal nuclease domain‐containing protein 1	*SND1*	<0.001	−4.12
	Isoleucine–tRNA ligase, cytoplasmic	*IARS*	0.002	−3.97
	Eukaryotic translation initiation factor 5	*EIF5*	<0.001	−3.59
	Heterogeneous nuclear ribonucleoprotein H	*HNRNPH1*	0.007	−3.26
	Ras‐related protein Rab‐7a	*RAB7A*	0.002	−3.18
	Sec1 family domain‐containing protein 1	*SCFD1*	<0.001	−3.14
	Cytochrome b	*MT‐CYB*	0.013	−3.09
	Spectrin beta chain, brain 1	*SPTBN1*	0.006	−2.90
	Torsin‐1A‐interacting protein 1	*TOR1AIP1*	<0.001	−2.84
	60S ribosomal protein L26	*RPL26; RPL26L1*	<0.001	−2.81
	Protein disulfide‐isomerase	*P4HB*	<0.001	−2.77
	Synaptophysin‐like protein 1	*SYPL1*	<0.001	−2.68
	NADH dehydrogenase [ubiquinone] 1 alpha subcomplex subunit 9, mitochondrial	*NDUFA9*	<0.001	−2.61
	Neuroblastoma‐amplified sequence	*NBAS*	0.001	−2.52
	Mitotic checkpoint protein BUB3	*BUB3*	<0.001	−2.51
	cAMP‐dependent protein kinase type II‐alpha regulatory subunit	*PRKAR2A*	<0.001	−2.49
	Disks large homolog 1	*DLG1*	<0.001	−2.44
	Centromere/kinetochore protein zw10 homolog	*ZW10*	0.001	−2.40
	Translin	*TSN*	0.001	−2.37
	Plakophilin‐2	*PKP2*	<0.001	−2.37
	Ras‐related protein Rab‐27B	*RAB27B*	<0.001	−2.29
	40S ribosomal protein S3a	*RPS3A*	<0.001	−2.21
	Tubulin alpha‐1A chain; Tubulin alpha‐3E chain	*TUBA1A; TUBA3E*	<0.001	−2.12
	LEM domain‐containing protein 2	*LEMD2*	<0.001	−2.05
	Eukaryotic translation initiation factor 3 subunit K	*EIF3K*	0.007	−2.03
	UMP‐CMP kinase	*CMPK1*	<0.001	−2.02

**TABLE 3:** Up‐regulated proteins (log FC ≥ 2) and down‐regulated proteins (log FC ≥ −2) in carcinoma ex‐pleomorphic adenoma compared to normal salivary glands.

	Protein name	Gene symbol	*p* Value	log_2_FC
Up‐regulated proteins	Decorin	*DCN*	<0.001	7.38
	Heterogeneous nuclear ribonucleoprotein L	*HNRNPL*	0.002	4.91
	Dystrophin	*DMD*	0.001	4.66
	Interferon regulatory factor 2‐binding protein‐like	*IRF2BPL*	<0.001	4.61
	Ras‐related protein Rab‐14	*RAB14*	0.003	4.21
	Coatomer subunit delta	*ARCN1*	<0.001	4.10
	Hsp90 co‐chaperone Cdc37	*CDC37*	0.003	4.03
	Bifunctional purine biosynthesis protein PURH	*ATIC*	<0.001	3.72
	Alcohol dehydrogenase [NADP(+)]	*AKR1A1*	<0.001	3.70
	FACT complex subunit SSRP1	*SSRP1*	0.006	3.70
	Aldehyde dehydrogenase family 16 member A1	*ALDH16A1*	0.009	3.54
	Tropomyosin beta chain	*TPM2*	0.009	3.05
	Hemoglobin subunit beta	*HBB*	0.003	3.04
	60S ribosomal protein L24	*RPL24*	0.004	3.02
	Protein S100‐A11	*S100A11*	<0.001	2.95
	Ras‐related protein Rab‐2A	*RAB2A*	<0.001	2.91
	Calmodulin‐like protein 5	*CALML5*	0.009	2.88
	Myosin light polypeptide 6	*MYL6*	0.001	2.69
	Nucleolar protein 56	*NOP56*	0.001	2.67
	Aspartate–tRNA ligase, cytoplasmic	*DARS*	<0.001	2.57
	S‐methyl‐5‐thioadenosine phosphorylase	*MTAP*	0.003	2.57
	Mitochondrial 2‐oxoglutarate/malate carrier protein	*SLC25A11*	<0.001	2.56
	Annexin A6	*ANXA6*	0.002	2.48
	Dynamin‐1‐like protein	*DNM1L*	0.002	2.44
	Tropomyosin alpha‐1 chain	*TPM1*	0.001	2.34
	Cytochrome c oxidase subunit 4 isoform 1, mitochondrial	*COX4I1*	0.005	2.28
	T‐complex protein 1 subunit theta	*CCT8*	0.002	2.19
	Translocating chain‐associated membrane protein 1	*TRAM1*	<0.001	2.15
	60S ribosomal protein L34	*RPL34*	<0.001	2.14
	Protein transport protein Sec24A	*SEC24A*	<0.001	2.07
	Methyltransferase‐like protein 7A	*METTL7A*	0.002	2.03
Down‐regulated proteins	Biglycan	*BGN*	<0.001	−7.29
	Adenylyl cyclase‐associated protein 1; Adenylyl cyclase‐associated protein	*CAP1*	<0.001	−4.39
	Staphylococcal nuclease domain‐containing protein 1	*SND1*	<0.001	−4.27
	Eukaryotic translation initiation factor 5	*EIF5*	<0.001	−3.90
	Protein disulfide‐isomerase	*P4HB*	<0.001	−3.35
	Translin	*TSN*	0.001	−3.27
	NADH dehydrogenase [ubiquinone] 1 alpha subcomplex subunit 10, mitochondrial	*NDUFA10*	<0.001	−3.12
	Heterogeneous nuclear ribonucleoprotein H	*HNRNPH1*	0.007	−2.76
	Spectrin beta chain, brain 1	*SPTBN1*	0.006	−2.55
	Isoleucine–tRNA ligase, cytoplasmic	*IARS*	0.002	−2.36
	40S ribosomal protein S14	*RPS14*	0.005	−2.21
	Ras‐related protein Rab‐7a	*RAB7A*	0.002	−2.09
	Heat shock protein 105 kDa	*HSPH1*	<0.001	−2.07
	Histone H1.0	*H1F0*	0.003	−2.06
	NADH dehydrogenase [ubiquinone] 1 alpha subcomplex subunit 9, mitochondrial	*NDUFA9*	<0.001	−2.05
	60S ribosomal protein L26	*RPL26; RPL26L1*	<0.001	−2.01
	Plakophilin‐2	*PKP2*	<0.001	−2.00

### 3.3 Interaction network maps and functional enrichment

The PPI analysis between dysregulated proteins showed a total of 87 interactions with one separate group of interactions being represented by ribosomal components and proteins related to translation (Figure 2b).

GO annotation also indicated that translation‐related processes are over‐represented by the dysregulated proteins (Table S5) with most functioning as structural and binding proteins (Table S6) and the most involved cellular components being intracellular, mainly cytoplasmic (Table S7). KEGG pathway analysis showed that the dysregulated proteins were associated with four significantly enriched pathways (Table S8).

### 3.4 Immunohistochemical analysis

**FIGURE 3:**
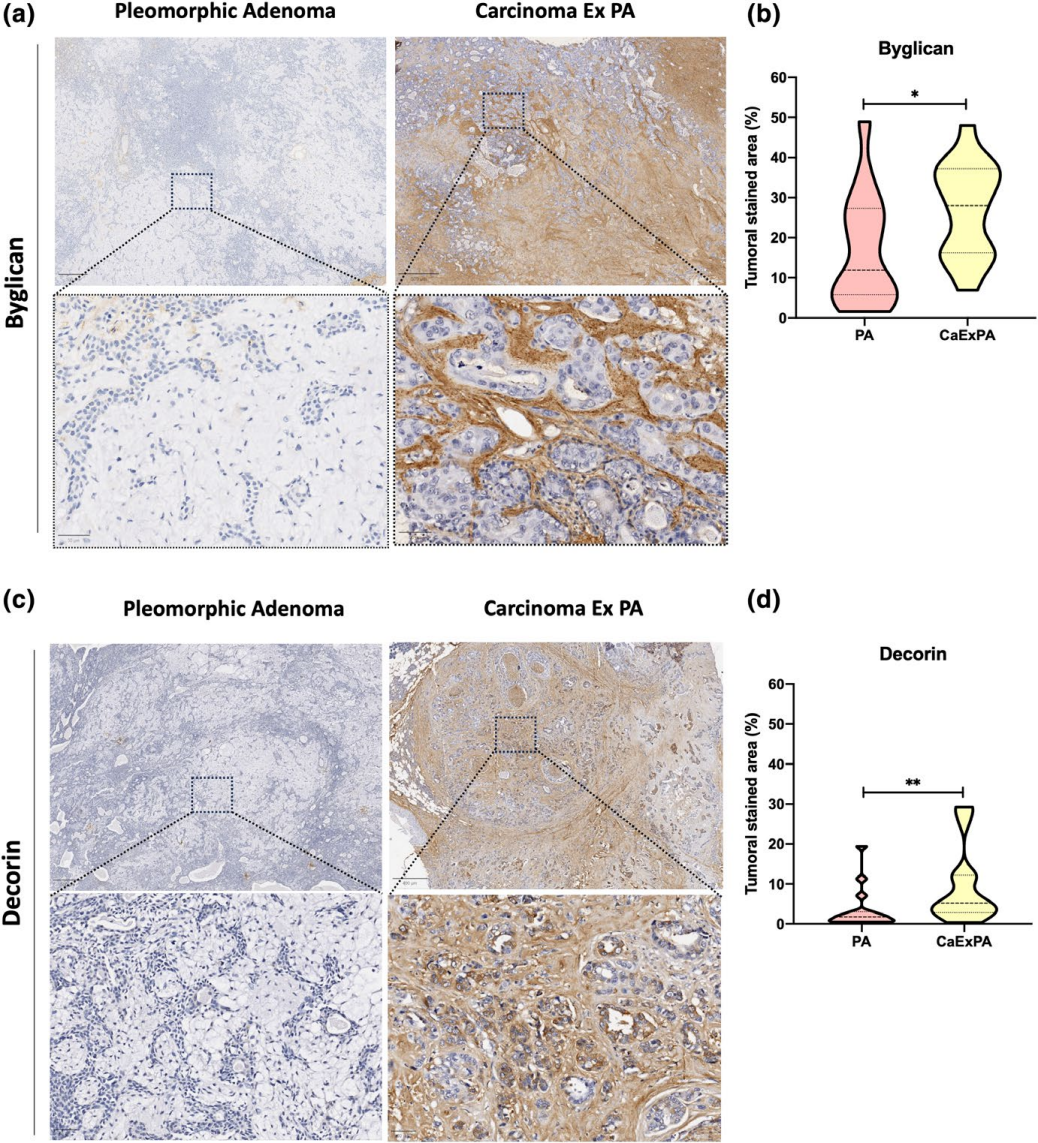
Representative images of low‐power and high‐power view of (a) Biglycan and (b) Decorin expression in pleomorphic adenoma and carcinoma ex‐pleomorphic adenoma. Statistical analysis confirmed that expression of (c) Biglycan and (d) decorin were higher in CXPA compared to PA (Mann–Whitney *U*‐test, *p < 0.05; **p < 0.01).

**FIGURE 4:**
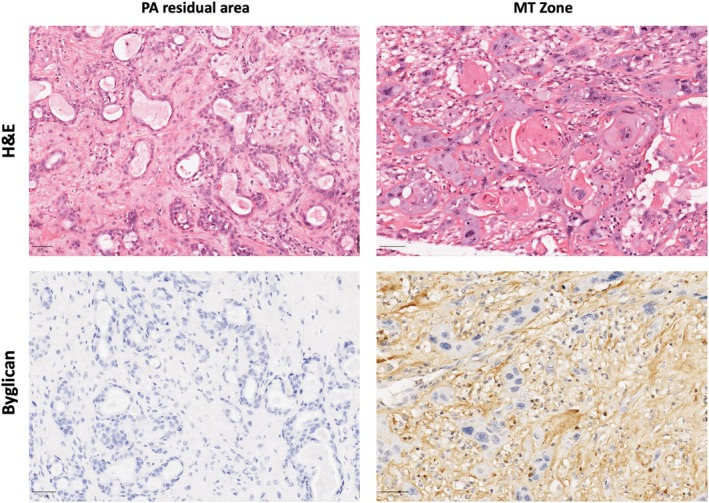
Representative image of Biglycan pattern of expression within one case of CXPA. Note that residual areas of PA showed absence of expression in the ECM component while areas of malignant transformation (characterized by bizarre pleomorphic cells) showed intense expression.

Due to the significant differences of Biglycan and Decorin levels between PA and CXPA, both proteins were selected for validation. Ductal cells from all three major NSG demonstrated Biglycan expression (Figure S3A) while in both the benign and malignant tumors the protein was almost exclusively expressed in the extracellular matrix (ECM) compartment, with both luminal and myoepithelial cells being negative (Figure 3a). Biglycan expression was significantly up‐regulated in CXPA compared to PA due to a more diffuse and more intense expression in the ECM compartment of malignant samples (*p* = 0.02, Figure 3b). It was also interesting to note that in five of the CXPA cases there was a very different pattern of expression between residual PA areas and areas of malignant transformation with the latter demonstrating Biglycan overexpression in comparison to absence of expression in the residual benign areas (Figure 4). No significant difference in Biglycan expression was seen when considering capsular invasion of CXPA cases (*p* = 0.54, Figure S4A).

Decorin was positive in the fibrous septa between lobules and surrounding ducts in NSG tissue, but was negative in ductal and acinar cells (Figure S3A). Benign samples showed almost no expression of Decorin (Figure 3c) with positivity only being observed in the fibrous capsule of the ECM compartment. CXPA demonstrated increased expression of Decorin compared to NSG and also in comparison to PA (*p* = 0.008, Figure 3d). In some CXPA cases, in addition to stromal expression, cytoplasmic staining was seen in malignant cells and Decorin was significantly overexpressed in widely invasive cases compared to intracapsular cases (*p* = 0.04, Figure S4B).

## 4 DISCUSSION

Proteomics can be used to identify deregulated biological mechanisms at the protein level in diseases, and as such has proved to be a useful tool in the search for diagnostic and prognostic markers for benign and malignant tumors. In the context of PA and CXPA, a very limited number of studies have been conducted using untargeted proteomic analysis (de Lima‐Souza et al., 2023; Mutlu et al., 2017), and this study is the first to include a range of samples that represent all the stages of CXPA carcinogenesis; from normal to benign tumor and finally its malignant counterpart. Our clustering analysis revealed that the proteome profile of PA and CXPA was significantly different from that of normal tissue. Significant differences in protein levels were also observed between benign and malignant tumors, for example, the up‐regulation of Translocation protein SEC63 homolog, Annexin A6 and Biglycan in CXPA compared to PA. This furthers our understanding and knowledge of the multi‐step process of PA and CXPA tumorigenesis. Due to the different levels of Biglycan and Decorin observed in PA and CXPA and based on our previous knowledge that the ECM plays an important role in their tumorigenesis, both proteins were selected for further validation in our study.

Comparison of our results to the literature is limited as, to the best of our knowledge and as previously mentioned, only one untargeted proteomic study has previously been conducted incorporating samples of PA and CXPA (de Lima‐Souza et al., 2023). In their study, samples from three groups (PA, residual PA, and CXPA) were analyzed to identify protein signatures and their results suggested that seven proteins (APOA1, AP1M1, SYCP1, DCD, HBB, HP, and SLC4A1) could be potential signatures for tumor progression or suppression. Interestingly, in our analysis, only HBB and SLC4A1 were detected with both being expressed in NSG, PA, and CXPA. While de Lima‐Souza et al. (2023) noted that HBB was up‐regulated only in PA compared to residual PA, in our study HBB was the third most up‐regulated protein in CXPA compared to PA (log_2_FC 3.7), and it was also up‐regulated in CXPA compared to NSG. We also found, again contrary to the findings of de Lima‐Souza et al. (2023) that there was no significant difference in SLC4A1 expression. As both studies only evaluated a limited number of cases the inconsistencies could be due to outliers within the cohort, limited area selected during LMS for analysis and/or due to differences in CXPA subtypes. Our study mostly comprised adenocarcinoma not otherwise specified (NOS) cases, while de Lima‐Souza et al. (2023) had four cases of each of the following CXPA subtypes: salivary duct carcinoma, myoepithelial carcinoma, and epithelial–myoepithelial carcinoma. Primary site might also have played a role in differences in expression seen as all of the PA and CXPA samples used for proteomics in the present study were primary parotid gland tumors while in the validation cohort, 72% of the CXPA cases originated in the parotid and the rest arising at varied sites. The primary site of cases included in the de Lima‐Souza et al. (2023) study is unclear, impeding our ability to draw any further conclusions.

In our study, the most up‐regulated protein in malignant cases was translocation protein SEC63 homolog which is encoded by the *SEC63* gene and plays an important role in the translocation of newly synthesized peptides into the ER (Linxweiler et al., 2017). In addition, proteins involved with translation‐related processes were found to be the most common dysregulated proteins based on our GO annotation analysis. Malignant cells proliferate at a higher rate than normal and benign cells and this enhanced metabolism demands continuous protein synthesis (Laham‐Karam et al., 2020). Based on this rationale, malignant cells could be susceptible to transcription and translation inhibitors which has led to initial studies and trials investigating the pharmacologically targeting of transcription through the use of Bromodomain inhibitors that can regulate the transcriptional machinery through epigenetic modifications and also drugs that can directly inhibit RNA polymerase (Laham‐Karam et al., 2020).

Our results also suggest a role for ECM‐related proteins in PA and CXPA tumorigenesis as the most differentially expressed proteins were Biglycan and Decorin. Few studies have previously investigated the expression of these proteoglycans in NSG or PA (Abiko et al., 1999; Zhao et al., 1999); however, these studies support our findings in that no Decorin expression was seen in PA but positive expression was noted in the fibrous septa of the adjacent non‐neoplastic salivary gland (Abiko et al., 1999; Zhao et al., 1999). Conflicting results have been described for Biglycan, as negligible expression was observed in the ECM component of PA in one study (Zhao et al., 1999) but in another study expression was described in PA neoplastic cells (Abiko et al., 1999). We observed weak expression in PA, but interestingly NSG ductal cells from all three major glands were positive for this protein, a finding not previously described. It is well established that Biglycan, apart from its ubiquitous role as an ECM structural component (Diehl et al., 2021), possesses the potential to function as a signaling molecule. Previous studies have confirmed a role for Biglycan in the innate immune system, where it has been shown to aggregate multiple receptor types and coordinate intricate signaling networks (Nastase et al., 2012). Our hypothesis is that ductal intra‐cellular expression might be associated with such roles.

In our proteomics analysis, Biglycan was significantly up‐regulated in CXPA compared to PA, and this was confirmed by immunohistochemical validation. Previous studies have suggested that Biglycan appears to have a dual role depending on the cancer type (Yu et al., 2023). For example, it has been associated with pro‐tumorigenesis and linked with increased proliferation, invasion, and metastasis in colorectal cancer, but it has also been described as having an anti‐proliferative role in pancreatic cancer cell lines (Diehl et al., 2021). Also, Biglycan can promote tumor angiogenesis through multiple mechanisms, including facilitating endothelial cell motility and stimulating the release of growth factors (Yu et al., 2023). Our results suggest that Biglycan increases during the final stages of CXPA tumorigenesis due to its global expression and our observation, through our immunohistochemical studies, of different patterns of expression in residual PA areas compared to malignant zones. Decorin, which is also an extracellular matrix (ECM) component (Diehl et al., 2021), was found in our proteomics analysis to be the most up‐regulated protein in both PA and CXPA in comparison to NSG. Our immunohistochemical results indicated lower expression than anticipated in the PA cases, potentially due to two outlier cases (both samples used for proteomic analysis; Table S8 and Figure S3B), which might have influenced the results, but also suggested that Decorin could be associated with the latter stages of CXPA tumorigenesis. Most of the previous data surrounding Decorin expression suggests a tumor‐suppressive role (Diehl et al., 2021); however, in CXPA it appears to have the opposite effect. Further studies are necessary to confirm this finding.

The most common molecular signatures of PA and CXPA are *PLAG1* rearrangements (Katabi et al., 2015; Voz et al., 2000) with *PLAG1* target genes being identified through microarray screening (Voz et al., 2004). Our proteomics data confirmed the presence of proteins encoded by some of these *PLAG1* target genes, namely *IGF2R, CRBAP2, SMARCE1, BCL2, LSP1, TGM2, PLEC, MAP4*, and *TNNT3*. Interestingly, ECM‐related genes are overrepresented among *PLAG1* targets (Voz et al., 2004), which corroborates our hypothesis that the ECM plays an important role in PA and CXPA tumorigenesis. A recent literature review discussed the role and importance of ECM components in PA and CXPA, including several proteoglycans such as aggrecan, perlecan, and tenascin (Scarini et al., 2023). The authors concluded that in CXPA, as with other cancers, dynamic changes in the tumor microenvironment, including the ECM compartment, play a role in malignant transformation. Therapies targeting these components could therefore prove beneficial, for example, it has been shown that Biglycan inhibition, through a nanodevice encapsulating a siRNA delivery system, significantly impairs tumor growth of xenografted renal cell carcinoma in nude mice (Maishi et al., 2022).

Although our results are novel and promising, we acknowledge that the relatively small sample size used for the proteomic step represents a limitation. This is illustrated in the fact that the immunohistochemistry analysis of Decorin in PA samples suggested a lower expression than was anticipated by the proteomics data. However, the validation of our proteomics findings does increase the strength of our findings and conclusions which significantly contribute to the very limited data currently available. To our knowledge, this is the first study to investigate the proteome of NSG, PA, and CXPA with a subsequent validation step. Another limitation is that we did not use paired samples; the PA and CXPA cases were not from the same patients. Proteomic analysis of samples from the same patients at different stages of malignant transformation would further enhance our understanding of the process. It is also important to highlight the challenge inherent in studying CXPA, which extends beyond its rarity to encompass the heterogeneity of the samples.

## 5 CONCLUSIONS

Untargeted mass spectrometry revealed that CXPA has higher levels of translocation protein SEC63 homolog, Annexin A6 and Biglycan in comparison to PA. Decorin was the most up‐regulated protein in neoplastic samples compared to NSG and immunohistochemistry validation using a larger sample cohort revealed that not only Biglycan but also Decorin were over‐expressed in CXPA compared to PA. These results suggest that proteins from the ECM play an important role in PA and CXPA tumorigenesis, with Biglycan and Decorin being associated with malignancy.

## AUTHOR CONTRIBUTIONS

**Virgílio Gonzales Zanella:** Methodology; investigation; formal analysis; writing – review and editing; data curation. **Sara Ferreira Dos Santos Costa:** Investigation; methodology; software; formal analysis; writing – review and editing. **Lauren Frenzel Schuch:** Methodology; formal analysis; investigation; writing – original draft. **Emily Ferreira Salles Pilar:** Investigation; methodology; writing – review and editing. **Adriana Franco Paes Leme:** Conceptualization; methodology; software; formal analysis; writing – review and editing. **Jean Nunes dos Santos:** Methodology; formal analysis; writing – review and editing. **Syed Ali Khurram:** Methodology; formal analysis; writing – review and editing; data curation; validation. **Fatima Elalawy:** Methodology; investigation; formal analysis; writing – review and editing; validation. **Lynne Bingle:** Methodology; formal analysis; writing – review and editing; investigation; validation. **Fabio Daumas Nunes:** Conceptualization; methodology; writing – review and editing. **Felipe Paiva Fonseca:** Conceptualization; methodology; formal analysis; writing – review and editing. **Pablo Agustin Vargas:** Conceptualization; methodology; writing – review and editing. **Manoela Domingues Martins:** Conceptualization; methodology; funding acquisition; supervision; resources; data curation; project administration; writing – original draft. **Vivian Petersen Wagner:** Writing – original draft; conceptualization; methodology; supervision; investigation; visualization; formal analysis.

## FUNDING INFORMATION

This study was funded by the Research Group of Hospital de Clínicas de Porto Alegre (DIPE/HCPA: 2021‐0634), Minas Gerais State Research Foundation (FAPEMIG); Coordination for the Improvement of Higher Education Personnel (CAPES, code 001); and the National Council for Scientific and Technological Development (CNPq).

## CONFLICT OF INTEREST STATEMENT

The authors declare no conflict of interest.

## Supporting information


Figures S1–S4

Table S1

Tables S2–S4

Tables S5–S8

## Data Availability

The data supporting the findings of this study are available within the supplementary table accompanying the manuscript. Researchers interested in obtaining the raw data should contact the corresponding author for assistance.
