# Supplementary material for: Pleomorphic adenoma and carcinoma ex‐pleomorphic adenoma tumorigenesis: A proteomic analysis
Source: Oral Dis. 2024 Aug 18;31(3):865–78. doi: 10.1111/odi.15109 (PMC12021308; doi:10.1111/odi.15109)
Supplement: Supplementary file 1 — Figures S1–S4 [file ODI-31-865-s001.zip › FiguresS1-S4_Legends.docx]

Figure S1. Representative image demonstrating (A) manual annotation area for further (B) pixel threshold analysis of DAB-stained area using QuPath software.

Figure S2. Histological classification of tumors. Representative images of (A) myxoid PA; (B) conventional PA and (C) cellular PA. Representative images of CXPA with malignant transformation to (D) myoepithelial carcinoma; (E) adenocarcinoma NOS; (F) salivary duct carcinoma and (F) epithelial-myoepithelial carcinoma. Representative images of a CXPA case showing (H) PA residual areas and (I) zones of malignant transformation.

Figure S3. (A) Biglycan and Decorin pattern of expression in all three major salivary glands. (B) Quantitative analysis of Biglycan and Decorin in normal salivary glands.

Figure S4. (A) Biglycan expression according to extent of capsular invasion in CXPA cases. (A) Decorin expression according to extent of capsular invasion in CXPA cases.
