# Supplementary figures and images for: Pleomorphic adenoma and carcinoma ex‐pleomorphic adenoma tumorigenesis: A proteomic analysis
Source: Oral Dis. 2024 Aug 18;31(3):865–78. doi: 10.1111/odi.15109 (PMC12021308; doi:10.1111/odi.15109)

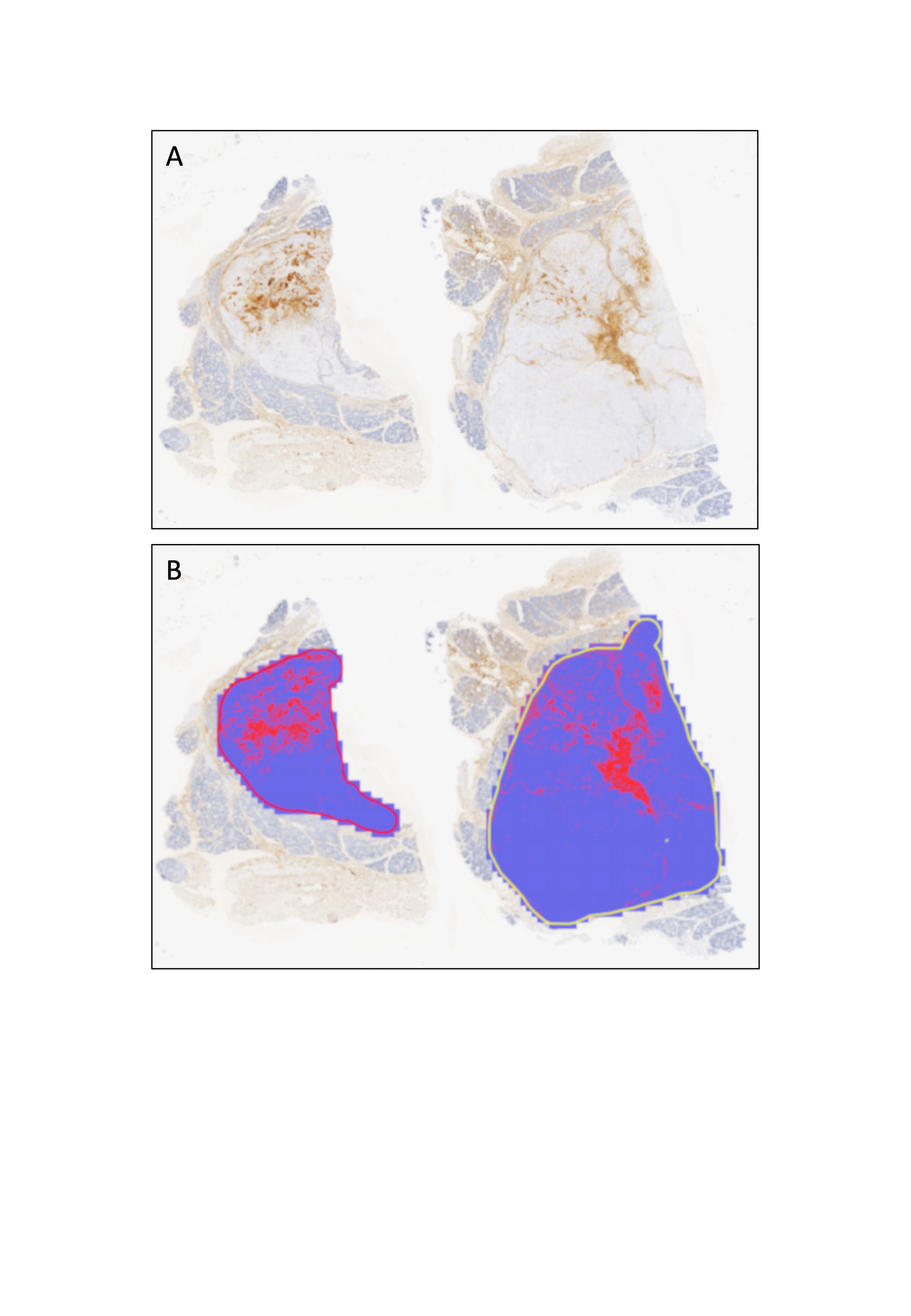

Supplement: Supplementary file 1 — Figures S1–S4 [file ODI-31-865-s001.zip › odi15109-sup-0001-FigureS1.tiff]

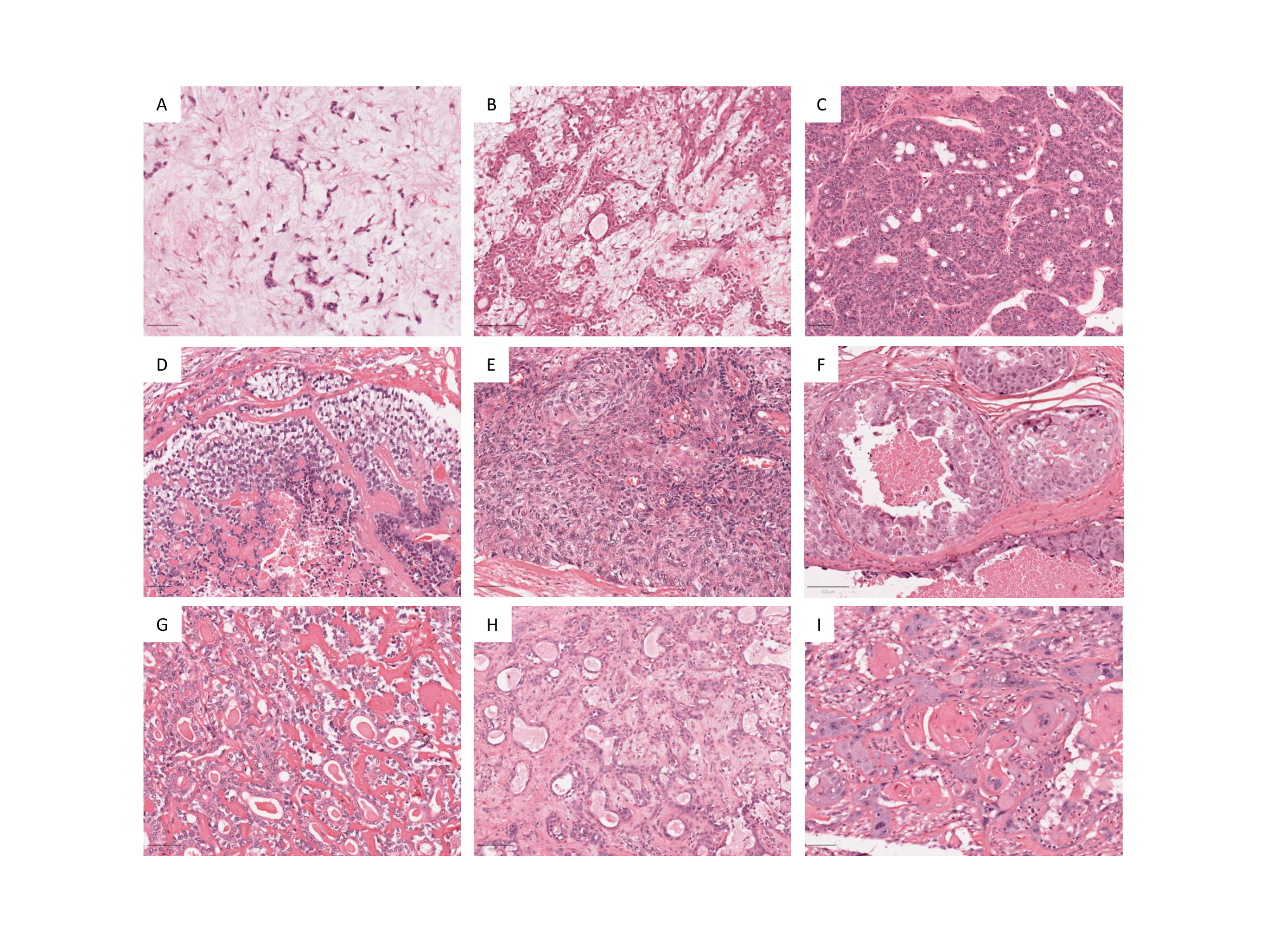

Supplement: Supplementary file 1 — Figures S1–S4 [file ODI-31-865-s001.zip › odi15109-sup-0002-FigureS2.tiff]

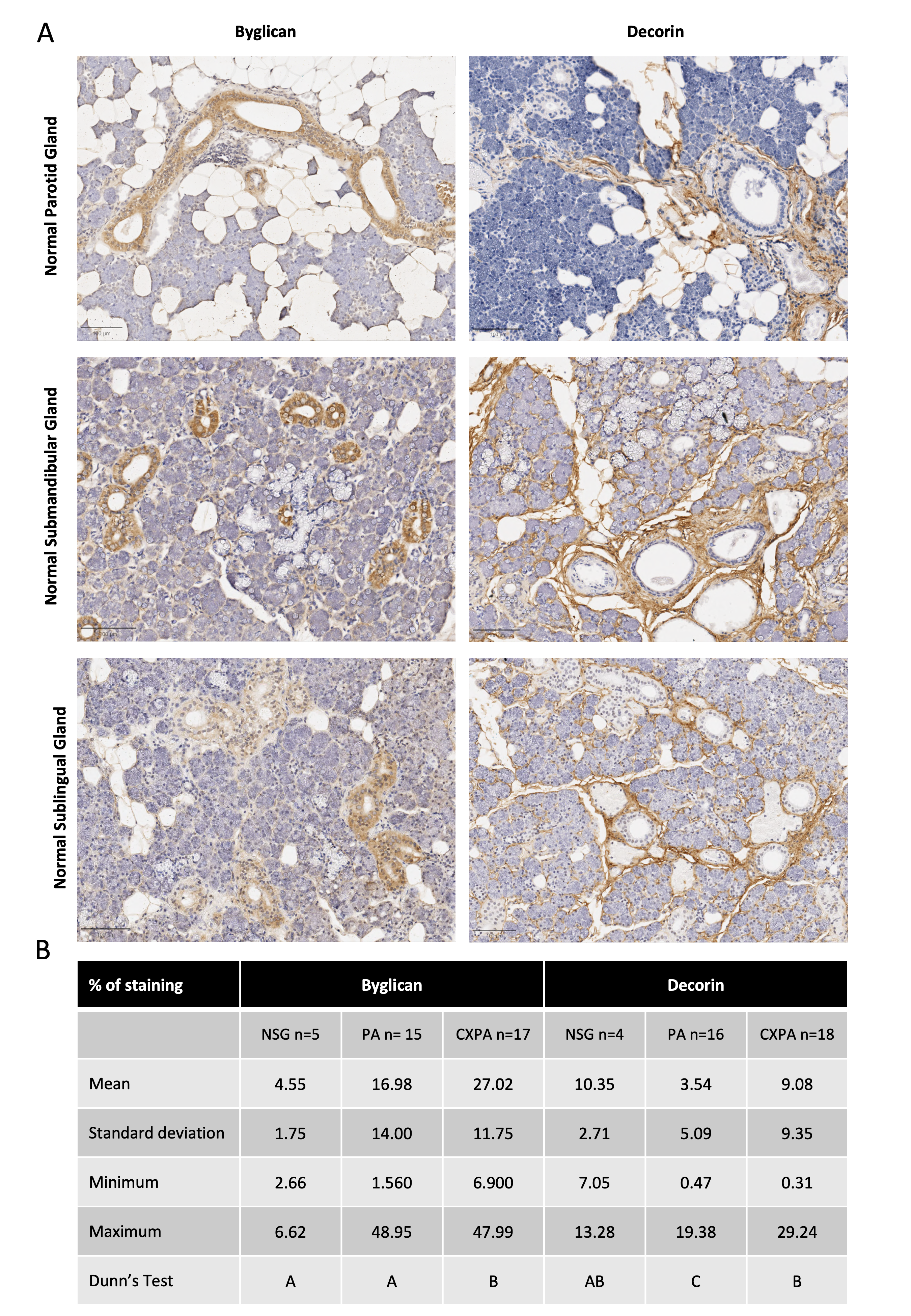

Supplement: Supplementary file 1 — Figures S1–S4 [file ODI-31-865-s001.zip › odi15109-sup-0003-FigureS3.tiff]

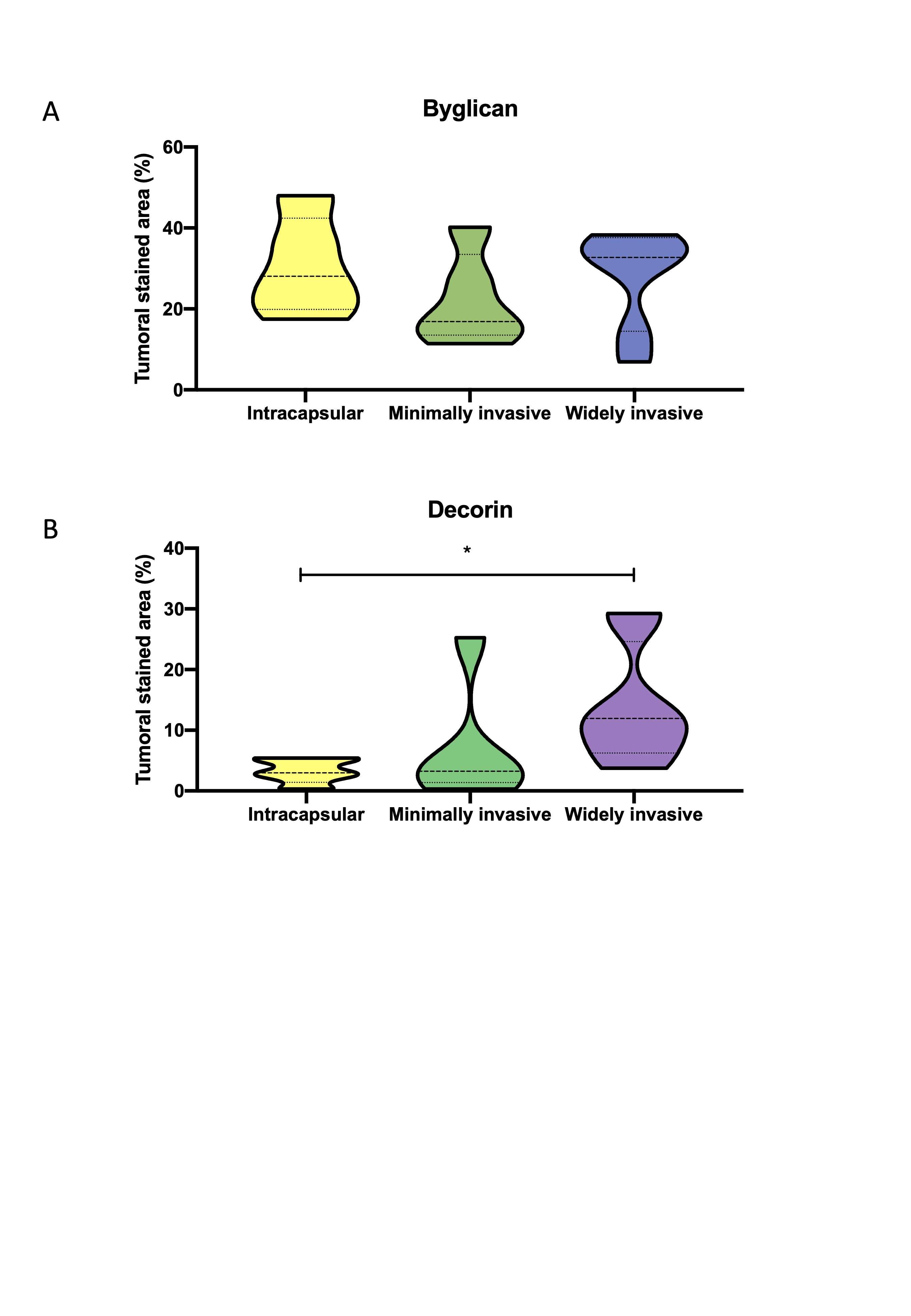

Supplement: Supplementary file 1 — Figures S1–S4 [file ODI-31-865-s001.zip › odi15109-sup-0004-FigureS4.tiff]
